# Gender-specific determinants of asthma among U.S. adults

**DOI:** 10.1186/s40733-017-0030-5

**Published:** 2017-01-24

**Authors:** Rebecca Greenblatt, Omar Mansour, Edward Zhao, Michelle Ross, Blanca E Himes

**Affiliations:** 1 Department of Biostatistics and Epidemiology, University of Pennsylvania, 219 Blockley Hall, 423 Guardian Drive, Philadelphia, PA 19104 USA; 2 Bloomberg School of Public Health, Johns Hopkins University, Baltimore, MD 21218 USA

**Keywords:** Adult asthma, Asthma, Obesity, Smoking

## Abstract

**Background:**

Asthma, a chronic respiratory disease affecting over 18.7 million American adults, has marked disparities by gender, race/ethnicity and socioeconomic status. Our goal was to identify gender-specific demographic and socioeconomic determinants of asthma prevalence among U.S. adults using data from the Behavioral Risk Factors Surveillance System (BRFSS) and the National Health and Nutrition Examination Survey (NHANES).

**Methods:**

Gender-specific regression analyses were performed to model the relationship between asthma prevalence with age, race/ethnicity, income, education level, smoking status, and body mass index (BMI), while taking into account the study designs.

**Results:**

Based on BRFSS data from 1,003,894 respondents, weighted asthma prevalence was 6.2% in males and 10.6% in females. Asthma prevalence among *grade 2 obese* and *grade 3 obese* vs. *not overweight or obese* women was 2.5 and 3.5 times higher, respectively, while that in men was 1.7 and 2.4 times higher; asthma prevalence among *current* vs. *never smoker* women was 1.4 times higher, while that in men was 1.1 times higher. Similar results were obtained with NHANES data from 13,364 respondents: asthma prevalence among *grade 2 obese* and *grade 3 obese* vs. *not overweight or obese* respondents was 2.0 and 3.3 times higher for women, though there was no significant difference for men; asthma prevalence among *current* vs. *never smokers* was 1.8 times higher for women and not significantly different in men. Asthma prevalence by race/ethnicity and income levels did not differ considerably between men and women.

**Conclusions:**

Our results underscore the importance of obesity and smoking as modifiable asthma risk factors that most strongly affect women.

**Electronic supplementary material:**

The online version of this article (doi:10.1186/s40733-017-0030-5) contains supplementary material, which is available to authorized users.

## Background

Asthma, a chronic obstructive lung disease characterized by variable airflow limitation and airway hyperresponsiveness, affects over 25.7 million Americans, including 18.7 million adults [1]. Women are disproportionately affected by asthma and adults are more likely to die from asthma than children [2]. The mechanisms underlying the gender difference in asthma prevalence are not known but hormonal and/or lung capacity differences between men and women have been proposed as contributing factors [3, 4]. While gender is not a modifiable risk factor, a better understanding of gender-specific factors that lead to increased asthma in women would result in therapies or interventions that reduce disparities.

Asthma prevalence and exacerbation disparities by race/ethnicity and socioeconomic status are also a well-known problem in the US [5–7]. Factors contributing to them include non-modifiable factors such as differences in genetic ancestry [8–10], and modifiable factors such as poor health, disease risk factors, medication non-adherence, health literacy and limited access to health care due to social, economic and environmental disadvantages [6, 11, 12]. Racial/ethnic minority groups in the U.S. disproportionately fall within low socioeconomic status categories, resulting in difficulty separating health factors that are heritable vs. socioeconomic and environmental ones [13, 14]. According to 2013 American Community Survey data, while racial/ethnic minorities made up 40% of all working families, they accounted for 58% of low-income families, and were twice as likely to be poor or low-income compared to non-Hispanic whites [15]. Understanding the contribution of modifiable vs. non-modifiable factors that influence asthma is challenging but important to reduce disease disparities.

The Behavioral Risk Factors Surveillance System (BRFSS) is a cross-sectional telephone survey of individuals greater than 18 years old that has collected data for over 30 years to measure behavioral risk factors that link to chronic and preventable diseases [16]. The survey is conducted by state health departments in conjunction with the Centers for Disease Control & Prevention (CDC) and includes the 50 U.S. states, the District of Columbia, and the U.S. territories of Guam, Puerto Rico, and the Virgin Islands. BRFSS data, whose quality is high, reliable and valid [17, 18], has been used to study disease trends and factors related to them, including obesity [19], cigarette smoking [20], binge drinking [21], and influenza vaccination rates [22]. Previous studies have also used BRFSS data to study asthma by identifying associated demographic factors with data from the years 2000 and 2009-2010 [23, 24], and finding that frequent mental distress [25] and poor self-rated physical and mental health [26] are more common among respondents with asthma than those without it.

The National Health and Nutrition Examination Survey (NHANES) is a CDC-led cross-sectional study designed to assess the health and nutritional status of adults and children in the U.S [27]. Highly trained personnel conduct extensive in-person household interviews and perform physical exams in mobile examination centers to obtain detailed information for enrolled subjects. Recruitment takes place via a complex probability cluster design to ensure samples are representative of the civilian, noninstitutionalized U.S. population. NHANES data has been used to understand asthma by studying its relationship with variables such as eosinophil counts, obesity and response to poor air quality [28–30], and to obtain reference distribution curves for measures such as exhaled nitric oxide [31].

Here, we used BRFSS data of over 1 million individuals and NHANES data of 13,364 individuals for the years 2007-2012 to better understand gender-specific factors that are associated with asthma among U.S. adults.

## Methods

### Behavioral Risk Factors Surveillance System (BRFSS) Data

BRFSS county-level data for the years 2007 to 2012 was downloaded from the BRFSS website (http://www.cdc.gov/brfss/). For our primary analysis, we combined the 6 years of survey data. Respondents were classified as having asthma based on affirmative answers to both of the BRFSS questions 1) “Have you ever been told by a doctor, nurse, or other health professional that you had asthma?” and 2) “Do you still have asthma?” The *never* asthma group consisted of respondents who answered “no” to the first question. We used the following individual response variables to capture information about respondent demographics and socioeconomic factors: age, gender, race/ethnicity, education level, household income, body mass index (BMI), and smoking status. Race/ethnicity included respondents classified as *White, Black, American Indian/Alaskan Native, Asian/Pacific Islander,* and *Hispanic*. Education level was re-leveled into three categories: *less than high school, high school, some college or more*. Household yearly income was re-leveled into three categories: *<$25,000; $25,000 - $75,000; ≥$75,000*. Smoking status was re-leveled into three groups: *current smoker, former smoker, and never smoker*. BMI was re-leveled into five groups: *not overweight or obese* (<25.0 kg/m^2^), *overweight* (25.0 to < 30.0 kg/m^2^), *grade 1 obese* (30.0 to <35.0 kg/m^2^)*, grade 2 obese* (35.0 kg/m^2^ to < 40.0 kg/m^2^) and *grade 3 obese* (>40.0 kg/m^2^). Respondents who were less than 22 years of age were excluded to reduce bias in response to education level and yearly household income questions from those who would not have begun college or working due to young age.

### National Health and Nutrition Examination Survey (NHANES) Data

NHANES data for the years 2007-2008, 2009-2010, and 2011-2012 was obtained using the nhanesA R package (https://CRAN.R-project.org/package=nhanesA). Respondents were classified as having asthma based on affirmative answers to both of the NHANES questions 1) “Has a doctor or other health professional ever told you that you have asthma?” and 2) “Do you still have asthma?” The *never* asthma group consisted of respondents who answered “no” to the first question. We used the following individual response variables to capture similar information about respondents as was captured with BRFSS: age, gender, race/ethnicity, education level, poverty-to-income ratio (PIR), BMI, and smoking status. Race/ethnicity included respondents in all NHANES categories, though *Mexican* was integrated into *Other Hispanic* to be consistent with the BRFSS categories: *Non-Hispanic White, Non-Hispanic Black, Hispanic,* and *Other*. PIR was re-leveled into two categories: *income below poverty line: ≤1, income above poverty line: >1*. Education level, smoking status and BMI were re-leveled into the same groups that were used with BRFSS. Respondents who were less than 22 years of age were excluded from analysis.

### Statistical analysis

Statistical analyses were conducted in R [32]. To determine factors associated with asthma, we used logistic regression analyses. Crude odds ratios (ORs) were computed with complete cases for each variable individually. To determine gender-specific factors associated with asthma, adjusted ORs were computed with all potential risk factors included in separate models for female and male respondents. To obtain estimates that were generalizable to the U.S. population, weighted analyses were conducted using the R *survey* package with CDC-computed study-specific weights provided for each respondent. Collinearity was assessed by estimating variance inflation factors with the R *car* package.

## Results

We obtained BRFSS data for 1,316,013 respondents at least 22 years of age from datasets corresponding to years 2007-2012. Subjects who responded “yes” to the question “Have you ever been told by a doctor, nurse, or other health professional that you had asthma?” but “no” to “Do you still have asthma?” were excluded from analyses. Missing rates for most variables was low (<5%), except for yearly household income, which was missing in 13.14% of respondents [Table 1]. Subjects included in the study vs. subjects excluded due to missing data had statistically significant differences in all categories (*p* < 0.0001), however, the ratios for asthma, race/ethnicity, and smoking status levels were similar. Variables that were most differently distributed between study and excluded subjects were gender (59% females included vs. 71% excluded), education level (68% included vs. 59% excluded had *some college or more*), yearly household income (32% included vs. 26% excluded had *≥ $75,000*), BMI (36% included vs. 41% excluded were *not overweight or obese*), and age (29% included vs. 40% excluded were *65+* years old). We restricted further analyses to the 1,003,894 respondents with complete data.

**Table 1:** BRFSS Respondent Characteristics. Overall characteristics of complete cases used in analyses (i.e., Study Subjects) and those excluded due to missing data (i.e., Incomplete Cases) corresponding to BRFSS surveys from years 2007-2012. For each category, N (%) for raw data are shown

	Missing Responses	Study Subjects	Incomplete Cases
		(*N* = 1,003,894)	(*N* = 258,370)
Asthma	4,885 (0.39)		
No Asthma		910,446 (90.69)	228,298 (90.06)
Asthma		93,448 (9.31)	25,187 (9.94)
Gender	0 (0.00)		
Male		408,785 (40.72)	74,797 (28.95)
Female		595,109 (59.28)	183,573 (71.05)
Race/Ethnicity	49,411 (3.91)		
White		791,219 (78.81)	161,124 (77.11)
Black		94,962 (9.46)	20,136 (9.64)
American Indian/Alaskan Native		8,995 (0.90)	1,910 (0.91)
Asian/Pacific Islander		31,782 (3.17)	6,226 (2.98)
Hispanic		76,936 (7.66)	19,563 (9.36)
Education level	5,079 (0.40)		
Less than high school		22,554 (2.25)	12,388 (4.89)
High school		300,157 (29.90)	90,843 (35.87)
Some college or more		681,183 (67.85)	150,060 (59.24)
Yearly Household income (USD)	165,884 (13.14)		
< $25,000		253,845 (25.29)	30,061 (32.50)
$25,000-$75,000		424,416 (42.28)	38,185 (41.29)
≥ $75,000		325,633 (32.44)	24,240 (26.21)
Body Mass Index (BMI)	63,813 (5.06)		
Not overweight or obese		364,981 (36.36)	79,819 (41.03)
Overweight		370,007 (36.86)	68,578 (35.25)
Grade 1 obese		170,604 (16.99)	29,649 (15.24)
Grade 2 obese		61,508 (6.13)	10,193 (5.24)
Grade 3 obese		36,794 (3.67)	6,318 (3.25)
Smoking status	10,969 (0.87)		
Never smoked		533,717 (53.16)	139,077 (56.22)
Former smoker		310,507 (30.93)	73,486 (29.70)
Current smoker		159,670 (15.91)	34,838 (14.08)
Age	13,785 (1.09)		
22-34		118,250 (11.78)	28,139 (11.50)
35-44		159,672 (15.91)	29,622 (12.11)
45-54		211,528 (21.07)	41,017 (16.77)
55-64		225,722 (22.48)	48,606 (19.87)
65+		288,722 (28.76)	97,201 (39.74)

**Fig. 1:**
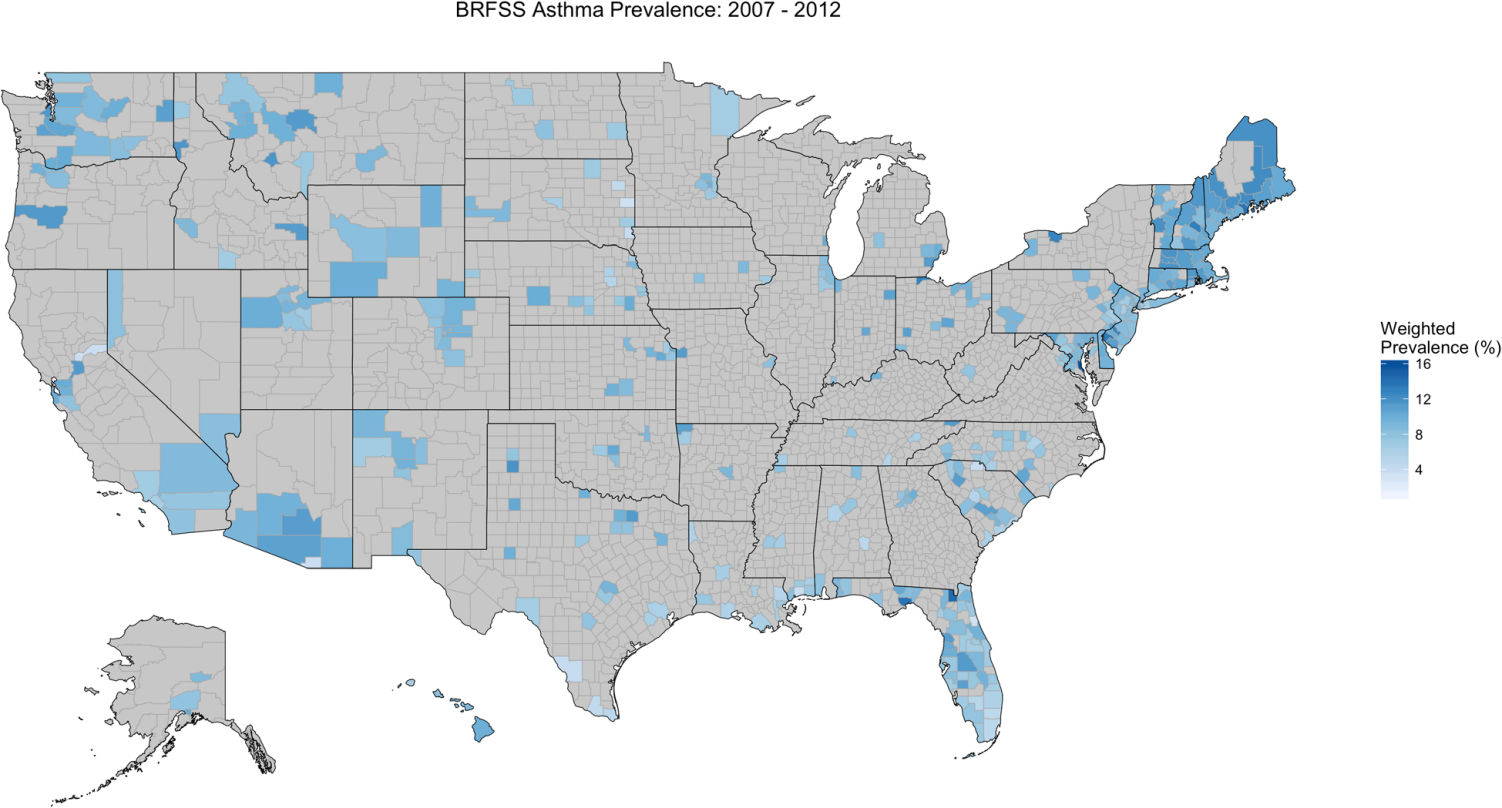
BRFSS U.S. Asthma Prevalence (2007-2012). Asthma was defined as affirmative responses to questions “Have you ever had asthma?” and “Do you currently have asthma?” Never asthma was based on negative response to “Have you ever had asthma?” Respondents were at least 22 years old at the time surveyed and resided in 408 U.S. counties. No data was available for counties shaded in grey

The weighted asthma prevalence for all respondents, who resided in 408 U.S. counties, was 8.41% [Fig. 1]. Asthma prevalence did not change greatly or monotonically over the 6-year span, ranging between 7.94% and 8.85% [see Table E1 in the Online Repository]. The demographic characteristics of respondents with asthma differed from those of never asthma respondents [Table 2]. From univariate analyses, asthma prevalence was increased in respondents who were female, obese, current or past smokers compared to never smokers, and had low yearly household income. Additionally, *black* and *American Indian/Alaskan Native* respondents had higher asthma prevalence relative to *white* respondents, while *Asian/Pacific Islander* and *Hispanic* respondents had lower asthma prevalence relative to *white* respondents. The percentages of respondents within categories in Table 2 and the crude ORs in Table 3 provide quantitative results illustrating such differences. Most notably, the *grade 2 obese* and *grade 3 obese* categories of BMI were strongly associated with asthma vs. *not overweight or obese* (crude OR 2.11; 95% CI: 2.00-2.23 and 3.30; 95% CI: 3.11-3.50, respectively); *female* vs. *male* gender had asthma crude OR 1.80 (95% CI: 1.74-1.86); and *< $25,000* vs. >*$75,000* yearly household income had asthma crude OR 1.60 (95% CI: 1.54-1.66).

**Table 2:** Asthma Prevalence by Demographic Categories. Overall characteristics of 2007-2012 BRFSS respondents according to asthma status. Counts provided (N) are for raw data. Weighted % are percentages obtained from analyzing BRFSS data using survey design with CDC-provided individual respondent weights, and thus, do not correspond directly to the raw counts. The weighted prevalence of asthma was 8.41%

	Asthma		Never Asthma	
	(*N* = 93,448)		(*N* = 910,446)	
	N	Weighted %	N	Weighted %
Gender				
Male	26,490	36.8	382,295	51.1
Female	66,958	63.2	528,151	48.9
Race/Ethnicity				
White	72,488	69.1	718,731	66.3
Black	10,643	14.4	84,319	11.7
American Indian/Alaskan Native	1,238	1.2	7,757	0.7
Asian/Pacific Islander	2,083	3.3	29,699	5.4
Hispanic	6,996	12.0	69,940	15.9
Education Level				
Less than high school	2,584	3.0	19,970	4.0
High school	30,212	30.8	269,945	29.1
Some college or more	60,652	66.2	620,531	66.9
Yearly Household Income (USD)				
< $25,000	33,218	31.5	220,627	23.5
$25,000-$75,000	36,290	37.6	388,126	39.6
≥ $75,000	23,940	30.9	301,693	36.9
Body Mass Index (BMI)				
Not overweight or obese	26,807	29.7	338,174	36.7
Overweight	29,952	32.8	340,055	37.8
Grade 1 obese	18,945	19.7	151,659	16.8
Grade 2 obese	9,535	9.7	51,973	5.6
Grade 3 obese	8,209	8.1	28,585	3.0
Smoking Status				
Never smoked	45,124	52.5	488,593	57.6
Former smoker	30,494	27.5	280,013	25.8
Current smoker	17,830	20.0	141,840	16.6
Age				
22-34	11,824	25.8	106,426	24.5
35-44	14,857	20.8	144,815	22.2
45-54	20,414	21.2	191,114	21.2
55-64	22,190	16.7	203,532	15.7
65+	24,163	15.5	264,559	16.5

**Table 3:** Factors Associated with Asthma Prevalence in BRFSS Univariate Analyses. Crude odds ratios (ORs) were derived from unadjusted survey logistic regression models with asthma as the outcome. Shown are ORs and 95% confidence intervals (CIs)

	Crude ORs (*N* =1,003,894)
Gender	
Male	Reference
Female	1.80 (1.74, 1.86)**
Race/Ethnicity	
White	Reference
Black	1.18 (1.13, 1.23)**
American Indian/Alaskan Native	1.64 (1.42, 1.90)**
Asian/Pacific Islander	0.59 (0.53, 0.65)**
Hispanic	0.72 (0.69, 0.76)**
Education level	
Less than high school	0.76 (0.69, 0.84)**
High school	1.07 (1.04, 1.10)**
Some college or more	Reference
Yearly Household income (USD)	
< $25,000	1.60 (1.54, 1.66)**
$25,000-$75,000	1.14 (1.10, 1.18)**
≥ $75,000	Reference
Body Mass Index (BMI)	
Not overweight or obese	Reference
Overweight	1.07 (1.03, 1.11)**
Grade 1 obese	1.45 (1.39, 1.51)**
Grade 2 obese	2.11 (2.00, 2.23)**
Grade 3 obese	3.30 (3.11, 3.50)**
Smoking status	
Never smoked	Reference
Former smoker	1.17 (1.13, 1.20)**
Current smoker	1.32 (1.27, 1.37)**
Age	
10 years	0.98 (0.97, 0.99)**

*p < 0.05, **p < 0.001

Due to the high crude OR of *female* compared to *male* gender, and the possible interaction of gender with other demographic and socioeconomic variables, we performed adjusted analyses for males and females separately to determine which factors were independently associated with asthma in a gender-specific fashion [Table 4]. Weighted asthma prevalence in males was 6.20% and in females was 10.62%. Asthma prevalence rates did not change greatly or monotonically over the 6-year span for either gender: the range was 5.69%-6.54% in males and 10.17%-11.22% in females [See Additional file 1: Tables E2 and E3 in the Online Repository]. We did not observe multi-collinearity between independent variables (all had generalized variance inflation factors < 2), suggesting that inclusion of all terms in regression models was appropriate.

**Table 4:** Gender-Specific Factors Associated with Asthma Prevalence in BRFSS Multivariate Analyses. Adjusted odds ratios (ORs) were derived separately for males and females from adjusted survey logistic regression models with asthma as the outcome. Shown are ORs and 95% confidence intervals (CIs)

	Male Adjusted ORs (*N* = 408,785)	Female Adjusted ORs (*N* = 595,109)
Weighted Asthma Prevalence (%)	6.20	10.62
Race/Ethnicity		
White	Reference	Reference
Black	0.97 (0.89, 1.06)	0.93 (0.89, 0.98)*
American Indian/Alaskan Native	1.36 (1.00, 1.86)	1.47 (1.28, 1.70)**
Asian/Pacific Islander	0.72 (0.61, 0.85)**	0.62 (0.54, 0.71)**
Hispanic	0.56 (0.50, 0.62)**	0.72 (0.67, 0.77)**
Education level		
Less than high school	0.76 (0.63, 0.93)*	0.66 (0.58, 0.74)**
High school	0.91 (0.85, 0.97)*	0.87 (0.83, 0.90)**
Some college or more	Reference	Reference
Yearly Household income (USD)		
< $25,000	1.61 (1.49, 1.74)**	1.53 (1.45, 1.60)**
$25,000-$75,000	1.09 (1.02, 1.15)*	1.07 (1.03, 1.12)**
≥ $75,000	Reference	Reference
Body Mass Index (BMI)		
Not overweight or obese	Reference	Reference
Overweight	1.01 (0.94, 1.08)	1.35 (1.29, 1.40)**
Grade 1 obese	1.22 (1.13, 1.33)**	1.82 (1.73, 1.91)**
Grade 2 obese	1.69 (1.52, 1.87)**	2.48 (2.33, 2.64)**
Grade 3 obese	2.35 (2.07, 2.68)**	3.49 (3.26, 3.73)**
Smoking status		
Never smoked	Reference	Reference
Former smoker	1.16 (1.09, 1.23)**	1.27 (1.23, 1.32)**
Current smoker	1.11 (1.03, 1.20)*	1.41 (1.34, 1.47)**
Age		
10 Years	0.94 (0.93, 0.96)**	0.95 (0.94, 0.97)**

*p < 0.05, **p < 0.001

In adjusted analyses, the effects of BMI and smoking status differed by gender [Table 4]. The adjusted ORs for asthma increased with increasing BMI more steeply in women than men. While *grade 2 obese* and *grade 3 obese* male respondents had a 1.69 and 2.35 times increased prevalence of asthma vs. *not overweight or obese* males (95% CI: 1.52-1.87 and 2.07-2.68), *grade 2 obese* and *grade 3 obese* female respondents had a 2.48 and 3.49 times increased prevalence of asthma vs. *not overweight or obese* females (95% CI: 2.33-2.64 and 3.26-3.73); g*rade 1 obese* males had a 1.22 times increased prevalence compared to *not overweight or obese* males (95% CI: 1.13-1.33) and *grade 1 obese* females had a 1.82 times increased prevalence compared to *not overweight or obese females* (95% CI: 1.73-1.91); *overweight* females had a 1.35 times increased asthma prevalence compared to their *not overweight or obese* counterparts (95% CI: 1.29-1.40), but *overweight* males did not have a significantly increased prevalence compared to their *not overweight or obese* counterparts. Females that were *current smokers* had a 1.41 times increased asthma prevalence compared to females that *never smoked* (95% CI: 1.34-1.47), and males that were *current smokers* had a 1.11 times increased prevalence compared to males that *never smoked* (95% CI: 1.03-1.20); female *former smokers* had a 1.27 times increased prevalence compared to their counterparts who *never smoked* (95% CI: 1.23-1.32), while male *former smokers* had a 1.16 times increased prevalence compared to their counterparts who *never smoked* (95% CI: 1.09-1.23)*.*

The effects of race/ethnicity, education, yearly household income, and age varied less by gender in adjusted analyses. *Black* race/ethnicity relative to *white* did not have a significant effect on asthma prevalence in males, but had a 0.93 times decreased prevalence in females (95% CI: 0.89-0.98); *American Indian/Alaskan Native* race/ethnicity had an increased prevalence in both genders: 1.36 times in males (95% CI: 1.00-1.86) and 1.47 times in females (95% CI: 1.28-1.70). *Asian/Pacific Islander* race/ethnicity had a decreased prevalence in both genders: 0.72 times in males (95% CI: 0.61-0.85) and 0.62 times in females (95% CI: 0.54-0.71); *Hispanic* race/ethnicity also had a decreased prevalence in both genders: 0.56 times in males (95% CI: 0.50-0.62) and 0.72 times in females (95% CI: 0.67-0.77). *Less than high school* education level was associated with a decreased asthma prevalence of 0.76 times in males (95% CI: 0.63-0.93) and 0.66 times in females (95% CI: 0.58-0.74) relative to *some college or more. <$25,000* yearly household income was associated with a 1.61 times increased asthma prevalence in males (95% CI: 1.49-1.74) and a 1.53 times increased asthma prevalence in females (95% CI: 1.45-1.60) relative to *>* *$75,000* yearly household income.

Results obtained for males and females for each of the six BRFSS yearly datasets showed consistency across individual years for association of factors with asthma [See Additional file 1: Tables E2 and E3 in the Online Repository]. The ORs of *American Indian/Alaskan Native* race/ethnicity category were the most variable across years, consistent with this category having fewer respondents than others, and hence, greater uncertainty in values reported. County-level characteristics based on complete cases used in the analysis are available at http://asthmamaps.org.

Similar results to those obtained with BRFSS data were obtained with NHANES. Overall weighted asthma prevalence for 2007-2012 was 8.22% according to NHANES vs. 8.41% according to BRFSS. Asthma prevalence was 6.23% for men and 10.02% for women according to NHANES, vs. 6.20% for men and 10.62% for women according to BRFSS. In adjusted analyses, the effects of BMI and smoking status also differed by gender in NHANES, and the adjusted ORs for asthma increased with increasing BMI more steeply in women than men [Table 5]. *Grade 2 obese* and *grade 3 obese* male respondents did not have a significant increased prevalence of asthma vs. their *not overweight or obese* counterparts, while *grades 2* and *grade 3 obese* female respondents had a 2.00 and 3.30 times increased prevalence of asthma vs. *not overweight or obese* females (95% CI: 1.47-2.72 and 95% CI: 2.19-4.97). Females that were *current smokers* had a 1.78 times increased asthma prevalence compared to females that *never smoked* (95% CI: 1.35-2.33), and males that were *current smokers* did not have a statistically significant difference in asthma prevalence compared to males that *never smoked.* The effects of race/ethnicity, education, PIR, and age varied less by gender.

**Table 5:** Gender-Specific Factors Associated with Asthma Prevalence in NHANES Multivariate Analyses. Adjusted odds ratios (ORs) were derived separately for males and females from adjusted survey logistic regression models with asthma as the outcome. Shown are ORs and 95% confidence intervals (CIs)

	Male Adjusted ORs (*N* = 6,476)	Female Adjusted ORs (*N* = 6,888)
Weighted Asthma Prevalence (%)	6.23	10.02
Race/Ethnicity		
White	Reference	Reference
Black	0.98 (0.73, 1.33)	0.95 (0.76, 1.18)
Hispanic	0.39 (0.26, 0.59)**	0.62 (0.46, 0.83)*
Other	0.80 (0.48, 1.35)	0.77 (0.49, 1.21)
Education level		
Less than high school	1.04 (0.76, 1.42)	0.86 (0.68, 1.10)
High school	0.81 (0.54, 1.23)	0.91 (0.70, 1.19)
Some college or more	Reference	Reference
Poverty income ratio		
≤ 1	1.70 (1.24, 2.34)*	1.78 (1.44, 2.20)**
> 1	Reference	Reference
Body Mass Index (BMI)		
Not overweight or obese	Reference	Reference
Overweight	0.91 (0.64, 1.29)	1.39 (1.06, 1.81)*
Grade 1 obese	1.18 (0.85, 1.63)	1.97 (1.54, 2.52)**
Grade 2 obese	1.69 (0.99, 2.89)	2.00 (1.47, 2.72)**
Grade 3 obese	1.74 (0.87, 3.48)	3.30 (2.19, 4.97)**
Smoking status		
Never smoked	Reference	Reference
Former smoker	1.34 (0.96, 1.87)	1.28 (1.00, 1.63)
Current smoker	1.27 (0.86, 1.89)	1.78 (1.35, 2.33)**
Age		
10 Years	1.03 (0.94, 1.12)	0.97 (0.90, 1.04)

*p < 0.05, **p < 0.001

## Discussion

Using 2007-2012 BRFSS data from 1,003,894 U.S. adult respondents, we quantified the association between asthma and socioeconomic and demographic factors. The strongest non-modifiable factor associated with asthma was gender, with women having an OR of 1.80 (95% CI 1.74-1.86) relative to men. We next performed gender-stratified BRFSS adjusted analyses to identify independent risk factors that varied in men and women. Similar analyses with NHANES adult data from 2007-2012 were performed to confirm BRFSS findings. While NHANES was a much smaller study (*n* = 13,364), some of its data is considered more reliable than that of BRFSS, as interviews were performed in-person and measures such as height and weight were obtained during physical exams, rather than via phone interviews.

The strongest association observed for asthma in both BRFSS and NHANES was with obesity, and this risk factor was particularly important among women. Our findings are consistent with the growing body of literature on the relationship between obesity and asthma, and more generally, metabolic syndrome and lung disease [33–36]. Prospective studies have found a dose-response relationship between the odds of asthma incidents and being overweight or obese [37, 38], and data from animal and human studies have shown that increases of normal adipose tissue lead to a systemic proinflammatory state [39]. Although the precise mechanism by which obesity contributes to asthma is not known, based on our findings and those of others, decreasing obesity should be a primary goal of interventions intended to reduce asthma. Our finding that obesity is a strong risk factor for asthma particularly in women is also consistent with a recent report based on U.S. National Health and Nutrition Examination Survey data, although in contrast with our results, that report found that obesity was not a significant risk factor in men [40]. The reason why obese women are at greater risk for asthma than obese men is not known.

Current smoking was another modifiable factor that was associated with asthma and had a greater effect in women than in men. The association between smoking and increased risk of asthma is well-documented, but there is little functional evidence that directly links smoking to asthma and it is unclear why the association is more pronounced in women than men [41, 42]. The gender-specific disparity observed between women and men was larger in NHANES than in BRFSS: women who were *current smokers* in NHANES had an OR of 1.78 for having asthma vs. *never smokers*, while those in BRFSS had an OR of 1.41. We are unable to determine whether this difference is due to unequal response reliability between the two studies or a reflection of cohort size differences.

While previous studies suggest that individuals differ in their genetic predisposition to asthma owing to differences in genetic ancestry that are reflected in racial/ethnic categories [8–10, 43], our BRFSS results suggest that other factors, such as income, more prominently lead to disparities in asthma prevalence by race/ethnicity. Although *black* and *American Indians/Alaskan Native* respondents had increased odds of asthma relative to *white* respondents in unadjusted analyses, after adjusting for other factors and stratifying by gender, the association with *black* race/ethnicity was not significant in males and led to decreased odds of asthma in females, and the association with *American Indians/Alaskan Natives* was considerably decreased in magnitude in both men and women. In NHANES adjusted analyses, statistically significant differences in asthma prevalence were observed for *Hispanic* men and women, who had the lowest prevalence of asthma among racial/ethnic groups.

The categorization of Hispanic respondents is limited, especially for BRFSS, in that we do not know respondent country of origin and large differences in asthma rates are known to occur among such groups: Mexican Americans have the lowest rate of asthma in the U.S. while Puerto Rican Americans have the highest rate [7]. Because BRFSS attempts to represent the U.S. population, the Hispanic results from this study most likely reflect responses from Mexican Americans, who according to the 2010 US census represent 63% of Hispanics in the U.S. Puerto Ricans, who are the next largest group, represent 9.2% of U.S. Hispanics [44]. NHANES included a *Mexican American* racial/ethnic category, and respondents who were classified in this group did have lowest asthma rates consistent with previous findings [7]: NHANES *Mexican American* males and females had adjusted ORs for asthma of 0.28 (95% CI: 0.17-0.45) and 0.35 (95% CI: 0.24-0.52), respectively, while NHANES *Other Hispanic* males and females had adjusted ORs for asthma of 0.59 (95% CI: 0.36-0.95) and 0.97 (95% CI: 0.71-1.32), respectively. Studies that gather more detailed information regarding Hispanic country of origin are likely to be more informative regarding the influence of demographic and socio-economic factors and asthma among U.S. Hispanics.

In BRFSS, low yearly household income (<*$25,000*) remained a significant risk factor for asthma in both genders after adjusting for other socioeconomic and demographic factors. Because a greater proportion of *black* and *American Indian/Alaskan Native* relative to *white* BRFSS respondents had low income, unadjusted analyses found stronger race/ethnicity associations within these groups. Our NHANES results also found that individuals of both genders living below the poverty line (PIR ≤1) were at increased risk of having asthma. The association between poverty and asthma has been observed and explored for many years [43, 45–47], and our results support the notion that individuals living in poverty experience a higher disease burden of asthma in the U.S. Continued studies that seek to understand the environmental and healthcare access factors, including gene-environment interactions [48], exposures (e.g., mold, cockroaches, and house dust mites) [49, 50] and health literacy [12, 51], that are associated with low income status and disproportionately affect some minority groups will decrease in asthma prevalence disparities by race/ethnicity.

Our findings are consistent with previous studies that used smaller portions of BRFSS data to examine demographic factors associated with asthma, although none performed gender-specific analyses [23, 24]. A study that used 2000 BRFSS data also found that obesity, female gender, current/past smoking, and low socioeconomic status were associated with asthma [24]. Another study that used 2009-2010 BRFSS data and performed race/ethnicity stratified analyses found that asthma risk factors differed by race/ethnicity [23]. For example, female gender and low income status were associated with asthma in all groups other than *Asian/Pacific Islanders*, and obesity was associated with asthma in all groups other than *American Indians/Alaskan Natives* [23].

By using NHANES and BRFSS data while accounting for their survey design, we obtained nationally representative measures from two independent studies with a large number of diverse participants. Although results from both studies were highly consistent, both datasets are subject to limitations. We were unable to make inferences about causality of observed associations due to the cross-sectional nature of BRFSS and NHANES. While the reliability and validity of BRFSS is high [17, 18], a study comparing it to other national surveys found that BRFSS asthma prevalence estimates were higher than those of the National Health Interview Survey (NHIS) [52]. BRFSS relies solely on self-reported data, and is thus, subject to error. Further, while we used a weighted survey design, residual bias from differences between survey respondents and the U.S. adult population may still affect our results. For example, BRFSS respondents were 60% female and 40% male, a distribution that is very skewed compared to the U.S. population. Comparison of other characteristics of our BRFSS respondents to those of the general U.S. population reveal other differences: (1) our study subjects consisted of 79% non-Hispanic White, 9% non-Hispanic Black, 8% Hispanic, 3% Asian/Pacific Islander and 1% American Indian/Alaskan Native respondents, while the 2010 U.S. Census reported a population of 64% non-Hispanic White, 13% Black, 16% Hispanic, 5% Asian/Pacific Islander and 1% American Indian/Alaskan Native [53], (2) 98% of our study subjects (all at least 22 years old) completed high school or more and 68% completed some college or more, while according to the American Community Survey, in 2015, 88% of the U.S. population at least 25 years old completed high school or higher and 59% had completed some college or more [54]. Household income of our study subjects was consistent with the U.S. mean of $55,775 in 2015 according to the American Community Survey [55], as 25% of our study subjects had a household income below $25,000, 42% had a household income between $25,000 and $75,000, and 32% had a household income above $75,000. The consistency we observed for BMI association results between BRFSS and NHANES suggests that self-reported measures of BMI from BRFSS were as reliable as those for NHANES, or that owing to its larger sample size, bias errors from BRFSS did not affect overall results for at least this variable. We used complete cases for our study, which may have introduced bias related to questions not answered by some groups although our sample size was large and included many subjects in most categories.

## Conclusions

Our results underscore the importance of obesity and smoking as asthma risk factors among US adults that impact women more strongly than men. To reduce asthma prevalence in adults overall, studies should continue to focus on identifying functional mechanisms by which women disproportionately develop asthma, as well as design interventions that effectively reduce obesity and smoking.

## Additional file

Additional file 1: This file contains three tables that provide yearly results of BRFSS data for 2007-2012. Table E1 contains results for all subjects. Tables E2 and E3 provide results for Male and Female subjects, respectively. (DOCX 161 kb)

## Abbreviations

BMI: Body mass index
BRFSS: Behavioral risk factors surveillance system
CDC: Centers for disease control & prevention
CI: confidence interval
FIPS: Federal Information Processing Standard
NHANES: National Health and Nutrition Examination Survey
OR: Odds ratio
