# Supplementary material for: Gender-specific determinants of asthma among U.S. adults
Source: Asthma Res Pract. 2017 Jan 24;3:2. doi: 10.1186/s40733-017-0030-5 (PMC5259982; doi:10.1186/s40733-017-0030-5)
Supplement: Additional file 1: — This file contains three tables that provide yearly results of BRFSS data for 2007-2012. Table E1 contains results for all subjects. Tables E2 and E3 provide results for Male and Female subjects, respectively. (DOCX 161 kb) [file 40733_2017_30_MOESM1_ESM.docx]

**Table E1.** Factors Associated with Asthma Prevalence for Each Year of BRFSS Data. Adjusted odds ratios (ORs) were derived from adjusted survey logistic regression models with asthma as outcome. Shown are weighted asthma prevalence estimates and ORs and 95% confidence intervals for each factor. *p<0.05, **p<0.001

|  |  | | **Adjusted ORs 2007 (N = 167,692)** | | **Adjusted ORs 2008 (N = 156,483)** | | **Adjusted ORs 2009 (N = 163,738 )** | |
| --- | --- | --- | --- | --- | --- | --- | --- | --- |
| **Weighted Asthma Prevalence (%)** | | | 7.94 | | 8.46 | | 8.13 | |
| Sex | | | | | | | | |
|  | Male | | Reference | | Reference | | Reference | |
|  | Female | | 1.92 (1.77, 2.08)** | | 1.66 (1.52, 1.81)** | | 1.76 (1.62, 1.90)** | |
| Race/Ethnicity | | | | | | | | |
|  | White | | Reference | | Reference | | Reference | |
|  | Black | | 0.91 (0.81, 1.02) | | 0.92 (0.82, 1.03) | | 0.86 (0.76, 0.97)* | |
|  | American Indian/Alaskan Native | | 1.54 (1.16, 2.04)* | | 2.02 (1.25, 3.28)* | | 1.35 (1.00, 1.81)* | |
|  | Asian/Pacific Islander | | 0.41 (0.31, 0.54)** | | 0.63 (0.49, 0.81)** | | 0.62 (0.49, 0.78)** | |
|  | Hispanic | | 0.62 (0.52, 0.73)** | | 0.55 (0.47, 0.65)** | | 0.56 (0.48, 0.65)** | |
| Education level | | | | | | | | |
|  | Less than high school | | 0.90 (0.65, 1.26) | | 0.59 (0.45, 0.77)** | | 0.78 (0.61, 1.01) | |
|  | High school | | 0.93 (0.85, 1.01) | | 0.83 (0.76, 0.91)** | | 0.94 (0.86, 1.03) | |
|  | Some college or more | | Reference | | Reference | | Reference | |
| Yearly Household income (USD) | | | | | | | | |
|  | <$25,000 | | 1.37 (1.22, 1.53)** | | 1.74 (1.56, 1.93)** | | 1.69 (1.52, 1.88)** | |
|  | $25,000-$75,000 | | 1.01 (0.92, 1.11) | | 1.08 (0.99, 1.17) | | 1.16 (1.06, 1.26)** | |
|  | ≥$75,000 | | Reference | | Reference | | Reference | |
| Body Mass Index (BMI) | | | | | | | | |
|  | Not overweight or obese | | Reference | | Reference | | Reference | |
|  | Overweight | | 1.29 (1.17, 1.42)** | | 1.26 (1.14, 1.39)** | | 1.21 (1.10, 1.32)** | |
|  | Grade 1 obese | | 1.54 (1.39, 1.72)** | | 1.68 (1.50, 1.88)** | | 1.51 (1.36, 1.68)** | |
|  | Grade 2 obese | | 2.10 (1.83, 2.41)** | | 2.43 (2.11, 2.80)** | | 2.08 (1.82, 2.37)** | |
|  | Grade 3 obese | | 3.16 (2.67, 3.73)** | | 3.20 (2.76, 3.71)** | | 2.98 (2.57, 3.45)** | |
| Smoking status | | | | | | | | |
|  | Never smoked | | Reference | | Reference | | Reference | |
|  | Former smoker | | 1.22 (1.12, 1.33)** | | 1.20 (1.10, 1.30)** | | 1.15 (1.06, 1.25)** | |
|  | Current smoker | | 1.18 (1.06, 1.30)* | | 1.22 (1.09, 1.35)** | | 1.18 (1.07, 1.30)* | |
| Age | 10 Years | | 0.94 (0.92, 0.97)** | | 0.95 (0.93, 0.98)** | | 0.93 (0.91, 0.95)** | |
|  |  | | **Adjusted ORs 2010 (N = 172,172)** | | **Adjusted ORs 2011 (N = 180,652)** | | **Adjusted ORs 2012 (N = 163,157 )** | |
| **Weighted Asthma Prevalence (%)** | | | | 8.85 | | 8.68 | | 8.48 |
| Sex | | | | | | | | |
|  | | Male | | Reference | | Reference | | Reference |
|  | | Female | | 1.87 (1.74, 2.01)** | | 1.80 (1.67, 1.95)** | | 1.90 (1.76, 2.06)** |
| Race/Ethnicity | | | | | | | | |
|  | | White | | Reference | | Reference | | Reference |
|  | | Black | | 0.96 (0.85, 1.08) | | 1.01 (0.91, 1.12) | | 1.00 (0.90, 1.11) |
|  | | American Indian/Alaskan Native | | 0.91 (0.67, 1.23) | | 1.34 (0.95, 1.90) | | 1.50 (1.12, 2.01)* |
|  | | Asian/Pacific Islander | | 0.94 (0.80, 1.11) | | 0.69 (0.54, 0.88)* | | 0.70 (0.52, 0.94)* |
|  | | Hispanic | | 1.04 (0.93, 1.17) | | 0.64 (0.56, 0.73)** | | 0.59 (0.51, 0.67)** |
| Education level | | | | | | | | |
|  | | Less than high school | | 0.52 (0.42, 0.63)** | | 0.88 (0.70, 1.12) | | 0.78 (0.60, 1.00) |
|  | | High school | | 0.85 (0.78, 0.91)** | | 0.87 (0.80, 0.95)** | | 0.93 (0.86, 1.01) |
|  | | Some college or more | | Reference | | Reference | | Reference |
| Yearly Household income (USD) | | | | | | | | |
|  | | <$25,000 | | 1.52 (1.39, 1.66)** | | 1.61 (1.46, 1.78)** | | 1.69 (1.53, 1.86)** |
|  | | $25,000-$75,000 | | 1.12 (1.03, 1.21)* | | 1.10 (1.01, 1.20)* | | 1.12 (1.02, 1.22)* |
|  | | ≥$75,000 | | Reference | | Reference | | Reference |
| Body Mass Index (BMI) | | | | | | | | |
|  | | Not overweight or obese | | Reference | | Reference | | Reference |
|  | | Overweight | | 1.24 (1.14, 1.35)** | | 1.18 (1.08, 1.29)** | | 1.16 (1.06, 1.27)* |
|  | | Grade 1 obese | | 1.65 (1.50, 1.81)** | | 1.57 (1.41, 1.73)** | | 1.56 (1.40, 1.75)** |
|  | | Grade 2 obese | | 2.22 (1.97, 2.49)** | | 2.05 (1.80, 2.35)** | | 2.20 (1.94, 2.50)** |
|  | | Grade 3 obese | | 3.25 (2.83, 3.73)** | | 2.76 (2.40, 3.17)** | | 3.12 (2.67, 3.65)** |
| Smoking status | | | | | | | | |
|  | | Never smoked | | Reference | | Reference | | Reference |
|  | | Former smoker | | 1.25 (1.16, 1.35)** | | 1.24 (1.14, 1.34)** | | 1.27 (1.17, 1.39)** |
|  | | Current smoker | | 1.30 (1.19, 1.43)** | | 1.40 (1.28, 1.54)** | | 1.42 (1.29, 1.56)** |
| Age | | 10 Years | | 0.96 (0.94, 0.98)** | | 0.95 (0.93, 0.97)** | | 0.94 (0.92, 0.97)** |

**Table E2.** Factors Associated with Asthma Prevalence for Each Year of BRFSS Data Among Males. Adjusted odds ratios (ORs) were derived from adjusted survey logistic regression models with asthma as outcome. Shown are weighted asthma prevalence estimates and ORs and 95% confidence intervals for each factor. *p<0.05, **p<0.001

|  |  | **Male Adjusted ORs 2007 (N = 65,750)** | **Male Adjusted ORs 2008 (N = 61,988)** | **Male Adjusted ORs 2009 (N = 65,656)** | |
| --- | --- | --- | --- | --- | --- |
| **Weighted Asthma Prevalence (%)** | | 5.69 | 6.54 | 6.06 |  |
| Race/Ethnicity | | | | | |
|  | White | Reference | Reference | Reference | |
|  | Black | 0.90 (0.71, 1.14) | 0.93 (0.75, 1.16) | 0.84 (0.66, 1.07) | |
|  | American Indian/Alaskan Native | 1.31 (0.77, 2.23) | 2.24 (0.87, 5.75) | 1.02 (0.60, 1.74) | |
|  | Asian/Pacific Islander | 0.46 (0.30, 0.73)** | 0.65 (0.44, 0.96)* | 0.80 (0.56, 1.13) | |
|  | Hispanic | 0.51 (0.37, 0.70)** | 0.42 (0.31, 0.57)** | 0.46 (0.34, 0.63)** | |
| Education level | | | | | |
|  | Less than high school | 1.14 (0.62, 2.09) | 0.66 (0.42, 1.04) | 1.02 (0.65, 1.60) | |
|  | High school | 0.93 (0.79, 1.10) | 0.88 (0.75, 1.03) | 0.98 (0.84, 1.15) | |
|  | Some college or more | Reference | Reference | Reference | |
| Yearly Household income (USD) | | | | | |
|  | <$25,000 | 1.27 (1.03, 1.56)* | 1.74 (1.43, 2.12)** | 1.88 (1.54, 2.29)** | |
|  | $25,000-$75,000 | 1.02 (0.87, 1.20) | 1.08 (0.93, 1.25) | 1.10 (0.95, 1.27) | |
|  | ≥$75,000 | Reference | Reference | Reference | |
| Body Mass Index (BMI) | | | | | |
|  | Not overweight or obese | Reference | Reference | Reference | |
|  | Overweight | 1.05 (0.89, 1.24) | 0.96 (0.80, 1.15) | 1.09 (0.92, 1.29) | |
|  | Grade 1 obese | 1.06 (0.88, 1.28) | 1.25 (1.00, 1.54)* | 1.35 (1.11, 1.64)* | |
|  | Grade 2 obese | 1.50 (1.17, 1.94)* | 1.71 (1.31, 2.24)** | 1.98 (1.53, 2.56)** | |
|  | Grade 3 obese | 2.14 (1.50, 3.05)** | 2.19 (1.55, 3.08)** | 3.08 (2.26, 4.19)** | |
| Smoking status | | | | | |
|  | Never smoked | Reference | Reference | Reference | |
|  | Former smoker | 1.32 (1.13, 1.55)** | 1.06 (0.92, 1.22) | 1.03 (0.90, 1.19) | |
|  | Current smoker | 1.08 (0.89, 1.31) | 0.93 (0.76, 1.13) | 0.96 (0.80, 1.16) | |
| Age | 10 Years | 0.94 (0.90, 0.98)* | 0.94 (0.90, 0.99)* | 0.91 (0.87, 0.95)** | |

|  |  | | **Male Adjusted ORs 2010 (N = 68,869)** | | **Male Adjusted ORs 2011 (N = 76,006)** | | **Male Adjusted ORs 2012 (N = 70,516)** |
| --- | --- | --- | --- | --- | --- | --- | --- |
| **Weighted Asthma Prevalence (%)** | | 6.45 | | 6.41 | | 6.07 | |
| Race/Ethnicity | | | | | | | |
|  | White | | Reference | | Reference | | Reference |
|  | Black | | 0.98 (0.79, 1.23) | | 1.15 (0.95, 1.39) | | 0.93 (0.77, 1.13) |
|  | American Indian/Alaskan Native | | 0.82 (0.45, 1.50) | | 1.33 (0.69, 2.59) | | 1.47 (0.88, 2.45) |
|  | Asian/Pacific Islander | | 0.99 (0.73, 1.34) | | 0.79 (0.54, 1.16) | | 0.62 (0.37, 1.04) |
|  | Hispanic | | 1.03 (0.83, 1.27) | | 0.59 (0.46, 0.75)** | | 0.46 (0.36, 0.59)** |
| Education level | | | | | | | |
|  | Less than high school | | 0.52 (0.36, 0.75)** | | 0.81 (0.53, 1.23) | | 0.96 (0.61, 1.51) |
|  | High school | | 0.90 (0.78, 1.04) | | 0.87 (0.75, 1.02) | | 0.94 (0.82, 1.09) |
|  | Some college or more | | Reference | | Reference | | Reference |
| Yearly Household income (USD) | | | | | | | |
|  | <$25,000 | | 1.51 (1.28, 1.79)** | | 1.71 (1.43, 2.03)** | | 1.79 (1.53, 2.10)** |
|  | $25,000-$75,000 | | 1.10 (0.96, 1.26) | | 1.13 (0.97, 1.31) | | 1.19 (1.02, 1.39)* |
|  | ≥$75,000 | | Reference | | Reference | | Reference |
| Body Mass Index (BMI) | | | | | | | |
|  | Not overweight or obese | | Reference | | Reference | | Reference |
|  | Overweight | | 1.03 (0.89, 1.20) | | 0.99 (0.84, 1.16) | | 0.92 (0.79, 1.07) |
|  | Grade 1 obese | | 1.30 (1.09, 1.55)* | | 1.20 (1.00, 1.45) | | 1.20 (0.97, 1.49) |
|  | Grade 2 obese | | 1.66 (1.32, 2.09)** | | 1.52 (1.18, 1.96)* | | 1.71 (1.36, 2.16)** |
|  | Grade 3 obese | | 2.29 (1.68, 3.11)** | | 1.99 (1.53, 2.59)** | | 2.46 (1.78, 3.40)** |
| Smoking status | | | | | | | |
|  | Never smoked | | Reference | | Reference | | Reference |
|  | Former smoker | | 1.16 (1.01, 1.34)* | | 1.16 (1.00, 1.33)* | | 1.26 (1.08, 1.46)* |
|  | Current smoker | | 1.15 (0.97, 1.37) | | 1.27 (1.08, 1.49)* | | 1.32 (1.12, 1.55)* |
| Age | 10 Years | | 0.97 (0.93, 1.01) | | 0.95 (0.91, 0.99)* | | 0.94 (0.90, 0.98)* |

**Table E3.** Factors Associated with Asthma Prevalence for Each Year of BRFSS Data Among Females. Adjusted odds ratios (ORs) were derived from adjusted survey logistic regression models with asthma as outcome. Shown are weighted asthma prevalence estimates and ORs and 95% confidence intervals for each factor. *p<0.05, **p<0.001

|  |  | **Female Adjusted ORs 2007 (N = 101,942)** | **Female Adjusted ORs 2008 (N = 94,495)** | **Female Adjusted ORs 2009 (N = 98,082)** | |
| --- | --- | --- | --- | --- | --- |
| **Weighted Asthma Prevalence (%)** | | 10.17 | 10.38 | 10.17 | |
|  |  |  |  |  | |
| Race/Ethnicity | | | | |  |
|  | White | Reference | Reference | Reference | |
|  | Black | 0.89 (0.78, 1.02) | 0.90 (0.79, 1.03) | 0.88 (0.77, 1.01) | |
|  | American Indian/Alaskan Native | 1.72 (1.24, 2.39)* | 1.83 (1.27, 2.63)* | 1.58 (1.10, 2.27)* | |
|  | Asian/Pacific Islander | 0.34 (0.24, 0.49)** | 0.59 (0.43, 0.81)* | 0.47 (0.35, 0.64)** | |
|  | Hispanic | 0.68 (0.55, 0.84)** | 0.65 (0.54, 0.78)** | 0.63 (0.54, 0.74)** | |
| Education level | | | | |  |
|  | Less than high school | 0.78 (0.55, 1.10) | 0.57 (0.40, 0.80)* | 0.67 (0.51, 0.89)* | |
|  | High school | 0.92 (0.83, 1.02) | 0.81 (0.73, 0.89)** | 0.92 (0.84, 1.02) | |
|  | Some college or more | Reference | Reference | Reference | |
| Yearly Household income (USD) | | | | |  |
|  | <$25,000 | 1.38 (1.21, 1.58)** | 1.69 (1.50, 1.91)** | 1.58 (1.41, 1.78)** | |
|  | $25,000-$75,000 | 0.99 (0.89, 1.11) | 1.06 (0.95, 1.17) | 1.18 (1.07, 1.31)* | |
|  | ≥$75,000 | Reference | Reference | Reference | |
| Body Mass Index (BMI) | | | | |  |
|  | Not overweight or obese | Reference | Reference | Reference | |
|  | Overweight | 1.40 (1.25, 1.56)** | 1.46 (1.31, 1.62)** | 1.28 (1.15, 1.42)** | |
|  | Grade 1 obese | 1.87 (1.65, 2.12)** | 1.97 (1.74, 2.22)** | 1.60 (1.43, 1.80)** | |
|  | Grade 2 obese | 2.44 (2.07, 2.87)** | 2.92 (2.49, 3.42)** | 2.09 (1.81, 2.42)** | |
|  | Grade 3 obese | 3.66 (3.03, 4.42)** | 3.76 (3.20, 4.42)** | 2.95 (2.52, 3.47)** | |
| Smoking status | | | | |  |
|  | Never smoked | Reference | Reference | Reference | |
|  | Former smoker | 1.17 (1.06, 1.29)* | 1.32 (1.20, 1.45)** | 1.23 (1.12, 1.36)** | |
|  | Current smoker | 1.24 (1.11, 1.39)** | 1.46 (1.29, 1.65)** | 1.33 (1.19, 1.48)** | |
| Age | 10 Years | 0.94 (0.91, 0.97)** | 0.96 (0.93, 0.99)* | 0.95 (0.92, 0.97)** | |

|  |  | | **Female Adjusted ORs 2010 (N = 103,303)** | | **Female Adjusted ORs 2011 (N = 104,646)** | | **Female Adjusted ORs 2012 (N = 92,641)** | |
| --- | --- | --- | --- | --- | --- | --- | --- | --- |
| **Weighted Asthma Prevalence (%)** | | 11.22 | | 10.99 | | 10.91 | |  |
|  |  | |  | |  | |  | |
| Race/Ethnicity | | | | | | | |  |
|  | White | | Reference | | Reference | | Reference | |
|  | Black | | 0.94 (0.81, 1.09) | | 0.92 (0.81, 1.04) | | 1.03 (0.91, 1.16) | |
|  | American Indian/Alaskan Native | | 0.96 (0.68, 1.37) | | 1.36 (0.97, 1.90) | | 1.50 (1.10, 2.06)* | |
|  | Asian/Pacific Islander | | 0.91 (0.75, 1.10) | | 0.61 (0.45, 0.82)* | | 0.75 (0.53, 1.07) | |
|  | Hispanic | | 1.06 (0.93, 1.20) | | 0.68 (0.58, 0.80)** | | 0.67 (0.58, 0.79)** | |
| Education level | | | | | | | |  |
|  | Less than high school | | 0.52 (0.41, 0.66)** | | 0.93 (0.70, 1.24) | | 0.67 (0.50, 0.90)* | |
|  | High school | | 0.82 (0.75, 0.89)** | | 0.87 (0.79, 0.95)* | | 0.92 (0.83, 1.01) | |
|  | Some college or more | | Reference | | Reference | | Reference | |
| Yearly Household income (USD) | | | | | | | |  |
|  | <$25,000 | | 1.50 (1.34, 1.66)** | | 1.51 (1.34, 1.70)** | | 1.59 (1.41, 1.80)** | |
|  | $25,000-$75,000 | | 1.11 (1.01, 1.22)* | | 1.06 (0.96, 1.18) | | 1.06 (0.95, 1.19) | |
|  | ≥$75,000 | | Reference | | Reference | | Reference | |
| Body Mass Index (BMI) | | | | | | | |  |
|  | Not overweight or obese | | Reference | | Reference | | Reference | |
|  | Overweight | | 1.35 (1.23, 1.48)** | | 1.29 (1.17, 1.43)** | | 1.31 (1.17, 1.47)** | |
|  | Grade 1 obese | | 1.86 (1.67, 2.08)** | | 1.84 (1.64, 2.07)** | | 1.81 (1.59, 2.05)** | |
|  | Grade 2 obese | | 2.55 (2.23, 2.91)** | | 2.45 (2.09, 2.86)** | | 2.49 (2.14, 2.90)** | |
|  | Grade 3 obese | | 3.74 (3.20, 4.35)** | | 3.25 (2.76, 3.81)** | | 3.49 (2.94, 4.16)** | |
| Smoking status | | | | | | | |  |
|  | Never smoked | | Reference | | Reference | | Reference | |
|  | Former smoker | | 1.31 (1.21, 1.43)** | | 1.30 (1.18, 1.42)** | | 1.29 (1.17, 1.42)** | |
|  | Current smoker | | 1.39 (1.25, 1.54)** | | 1.49 (1.34, 1.67)** | | 1.49 (1.33, 1.67)** | |
| Age | 10 Years | | 0.96 (0.94, 0.98)* | | 0.95 (0.92, 0.97)** | | 0.95 (0.93, 0.98)** | |
